# Supplementary figures and images for: The association between religious participation and memory among middle-aged and older adults: A systematic review
Source: PLoS One. 2023 Aug 18;18(8):e0290279. doi: 10.1371/journal.pone.0290279 (PMC10437981; doi:10.1371/journal.pone.0290279)

**S1 Appendix. Number of articles on religion and memory, published annually from year 1980 to 2022.**


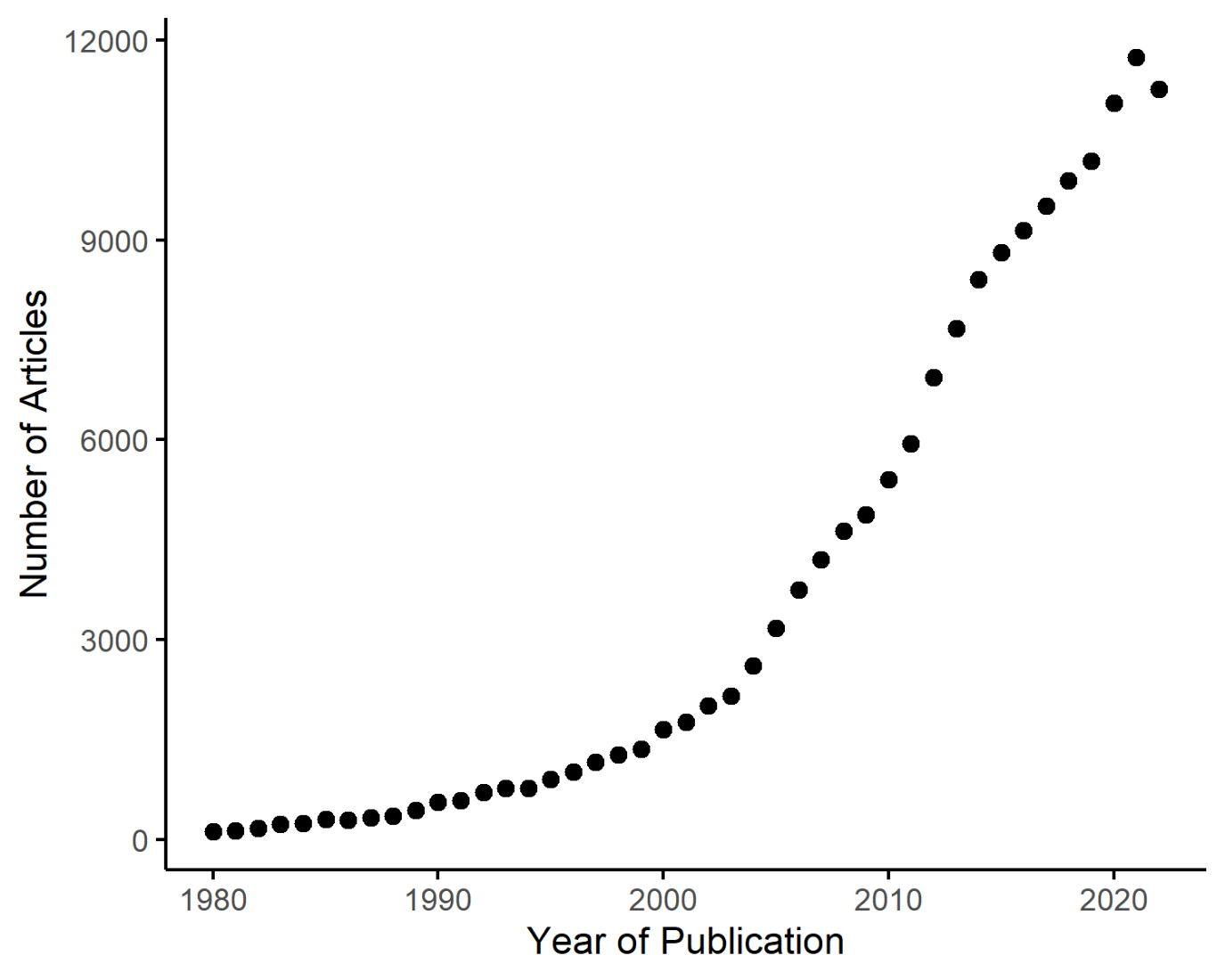

Supplement: S1 Appendix — (DOCX) [file pone.0290279.s001.docx]

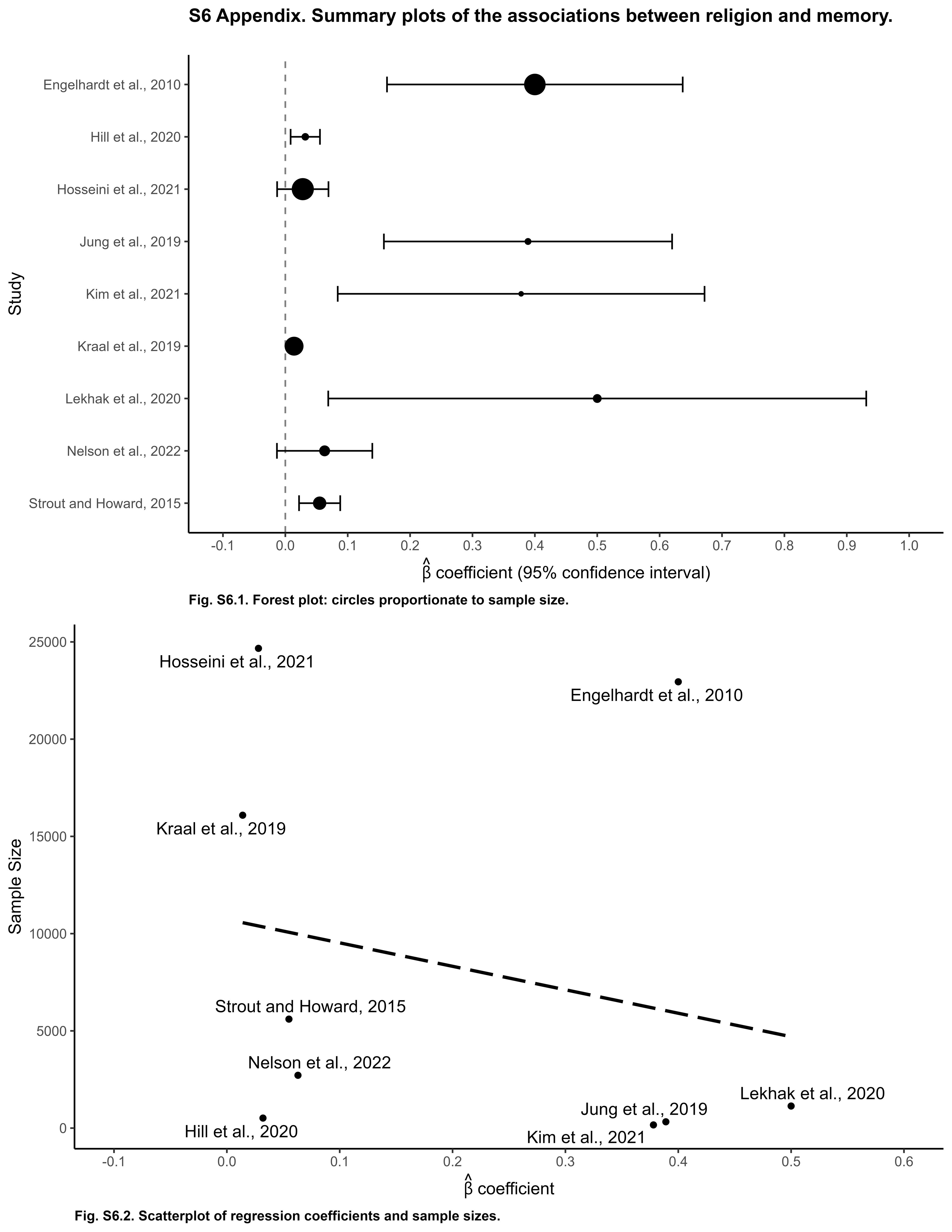

Supplement: S6 Appendix — (TIF) [file pone.0290279.s006.tif]
