# Supplementary material for: The association between religious participation and memory among middle-aged and older adults: A systematic review
Source: PLoS One. 2023 Aug 18;18(8):e0290279. doi: 10.1371/journal.pone.0290279 (PMC10437981; doi:10.1371/journal.pone.0290279)
Supplement: S3 Appendix — (DOCX) [file pone.0290279.s003.docx]

**S3 Appendix. Search phrases.**

**PsycINFO**

Any Field: Religious OR Any Field: Religion AND Any Field: cognitive function OR Any Field: cognitive aging OR Any Field: memory AND Any Field: depression OR Any Field: depressive OR Any Field: depressive disorders AND Any Field: older adults OR Any Field: elderly OR Any Field: old age OR Any Field: older people aged

**Scopus**

(TITLE-ABS-KEY (religion OR religious ) AND TITLE-ABS-KEY ( "Cognitive function" OR cognition OR memory OR "Cognitive aging" OR "cognitive reserve" ) AND TITLE-ABS-KEY ( "Older adult*" OR aged OR elderly OR "older people" OR "old age" OR geriatric) )

**PubMed**

(Religious or Religion) AND (cognitive function or cognitive aging or memory) AND (depression or depressive or depressive disorders) AND (older adults or elderly or old age or older people or aged)
