# Supplementary material for: The association between religious participation and memory among middle-aged and older adults: A systematic review
Source: PLoS One. 2023 Aug 18;18(8):e0290279. doi: 10.1371/journal.pone.0290279 (PMC10437981; doi:10.1371/journal.pone.0290279)
Supplement: S4 Appendix — (DOCX) [file pone.0290279.s004.docx]

**S4 Appendix. Synthesis Without Meta-analysis (SWiM) reporting items.**

| **SWiM** **is** **intended** **to** **complement** **and** **be** **used** **as** **an** **extension** **to** **PRISMA** | | | |
| --- | --- | --- | --- |
| **SWiM** **reporting**  **item** | **Item** **description** | **Page** **in** **manuscript**  **where** **item** **is** **reported** | **Other*** |
| ***Methods*** | | | |
| **1** Grouping studies for synthesis | 1a) Provide a description of, and rationale for, the groups used in the synthesis (e.g., groupings of populations, interventions, outcomes, study design). | 6 |  |
|  | 1b) Detail and provide rationale for any changes made subsequent to the protocol in the groups used in the synthesis. | NA |  |
| **2** Describe the standardised metric and transformation methods used | Describe the standardised metric for each outcome. Explain why the metric(s) was chosen, and describe any methods used to transform the intervention effects, as reported in the study, to the standardised metric, citing any methodological guidance consulted. | Pages 9-10 |  |
| **3** Describe the  synthesis methods | Describe and justify the methods used to synthesise the effects for each outcome when it was not possible to undertake a meta-analysis of effect estimates. |  | Cannot synthesize due to the heterogeneity of the studies. Instead, we narratively summarized the results. |
| **SWiM** **reporting**  **item** | **Item** **description** | **Page** **in** **manuscript**  **where** **item** **is** **reported** | **Other*** |
| **4** Criteria used to prioritize results for summary and synthesis | Where applicable, provide the criteria used, with supporting justification, to select the particular studies, or a particular study, for the main synthesis or to draw conclusions from the synthesis (e.g., based on study design, risk of bias assessments, directness in relation to the review question). |  | There were only 9 studies and all of them were narratively summarized. |
| **5** Investigation of heterogeneity in reported effects | State the method(s) used to examine heterogeneity in reported effects when it was not possible to undertake a meta-analysis of effect estimates and its extensions to investigate heterogeneity. | Pages 8-9 |  |
| **SWiM** **reporting**  **item** | **Item** **description** | **Page** **in** **manuscript**  **where** **item** **is** **reported** | **Other*** |
| **6** Certainty of evidence | Describe the methods used to assess certainty of the synthesis findings. |  | We were not able to assess certainty of the synthesis findings. The lack of longitudinal studies and articles about the association of faith and memory suggests that the certainty of evidence is low. |

| **SWiM** **reporting**  **item** | **Item** **description** | **Page** **in** **manuscript**  **where** **item** **is** **reported** | **Other*** |
| --- | --- | --- | --- |
| **7** Data presentation methods | Describe the graphical and tabular methods used to present the effects (e.g., tables, forest plots, harvest plots).  Specify key study characteristics (e.g., study design, risk of bias) used to order the studies, in the text and any tables or graphs, clearly referencing the studies included. | Page 14, FIG 2, S5 Appendix, S6 Appendix, S7 Appendix |  |
| ***Results*** | | | |
| **8** Reporting results | For each comparison and outcome, provide a description of the synthesised findings, and the certainty of the findings. Describe the result in language that is consistent with the question the synthesis addresses, and indicate which studies contribute to the synthesis. | Pages 10-14 |  |
| ***Discussion*** |  |  |  |
| **9** Limitations of the synthesis | Report the limitations of the synthesis methods used and/or the groupings used in the synthesis, and how these affect the conclusions that can be drawn in relation to the original review question. |  | Was not reported due to the heterogeneity of studies. |

PRISMA=Preferred Reporting Items for Systematic Reviews and Meta-Analyses.

NA=not applicable.

*If the information is not provided in the systematic review, give details of where this information is available (e.g., protocol, other published papers (provide citation details), or website (provide the URL) [27].
