# Supplementary material for: The association between religious participation and memory among middle-aged and older adults: A systematic review
Source: PLoS One. 2023 Aug 18;18(8):e0290279. doi: 10.1371/journal.pone.0290279 (PMC10437981; doi:10.1371/journal.pone.0290279)
Supplement: S5 Appendix — (DOCX) [file pone.0290279.s005.docx]

**S5 Appendix. Data extraction of included articles.**

| **Author, year** | **Study design** | **Study Characteristics (sample size, %female, mean age, country, source population, select exclusion criteria)** | **Religion/**  **Spirituality (exposure) Assessment** | **Memory (outcome) Assessment** | **Statistical test**  **Covariates** | **Results** |
| --- | --- | --- | --- | --- | --- | --- |
| Jung et al., 2019 | Cross-sectional | n=325  72.3% women  79.15±6.47 years  South Korea  Outpatients of psychiatric clinic  Exclusions: has major comorbidities (e.g., major depressive disorder, organic brain syndrome, vascular dementia), practices religion other than Christianity or Buddhism, scores more than 5 (moderately severe cognitive decline) on Global Deterioration Scale, has cognitive disorder caused by factors other than Alzheimer's disease according to Diagnostic and Statistical Manual of Mental Disorders, Fourth Edition | 3 scores derived from Duke University Religion Index (DUREL) subscales:  Organizational religious activity (ORA; frequency of attending religious meetings and other group-related religious activity)  Non organizational religious activity  (NORA; frequency of private religious activities such as prayer and meditation)  Intrinsic religiosity (IR; subjective experience about the Absolute, the importance of one’s belief, the strong will to carry on one’s belief)  Higher score = higher level of religiosity and spirituality | Single score derived from following Consortium to Establish a Registry for Alzheimer’s Disease Assessment Packet (CERAD) tests:  Word list memory test  Word list recall test  Word list recognition test  Constructional recognition test  Higher score=higher memory | Partial correlation analysis  Age, sex, years of education | ORA positively associated with memory (r=0.144, p=0.010)  NORA positively associated with memory (r=0.115, p=.040)  IR positively associated with memory (r=0.140, p=0.012) |
| Strout et al., 2015 | Cross-sectional | n=5,604  70% female  83±6.2 year  USA  Community-dwelling | Single score for spiritual wellness derived from Wellness assessment tool (WEL) | Single score derived using items from the following Cognitive Performance Scale (CPS) components:  Community health assessment (CHA)  Wellness assessment tool (WEL)  Functional supplement (FS)  Dichotomized to cognitively healthy (CPS score=0) or cognitively impaired (CPS score ≥1) for analyses 2 & 3  Higher score=higher impaired memory | 1. Hierarchical Multiple Regression Analysis  Age, education, social wellness, intellectual wellness, physical wellness, emotional wellness  2. discriminant analysis  3. Multivariate analysis of variance (Hotelling T2) | 1. Spiritual wellness negatively associated with impaired memory $\hat{\beta}$= −0.055, *p*<0.001  2. Standardized discriminant function coefficient for predictive power of spiritual wellness on cognitive impairment=0.311  3. Cognitively impaired group had statistically lower mean spiritual wellness scores than cognitively healthy group F(1,5,598)=38.430, p=0.0001 |

| Hill et al., 2020 | Cross-sectional | n=516  59% female  74.10±6.28 years  USA  Community-dwelling | 3 scores derived from:  Life-course religious attendance (more than once a week, once a week, 2 or 3 times a month, one or more times a year, less than once a year, or never): For before age 16, ages 16–29, and ages 30–49  Current religious attendance (more than once a week, once a week, 2 or more times a month, one or more times a year, or not at all)  Religious affiliation prior to age 16 ((1) Conservative Protestant (e.g., Baptist, Pentecostal, Assembly of God), (2) Mainline Protestant (e.g., Methodist, Lutheran, Presbyterian, Episcopal, Anglican), (3) other Protestant, (4) Catholic, (5) other religion (e.g., Mormon, Islam, Jewish), or (6) no religion.) | 3 scores derived from:  Self-rated memory  Immediate and delayed recall assessment to test episodic memory  Serial 7s assessment to test working memory  Higher scores=better memory | Ordinary least squares regression  Age, gender, educational attainment, race/ethnicity, employment status, wealth, early life relationship quality with mother, marital status, depressive symptoms, self-rated health | Life-course religious attendance negatively associated with working memory ($\hat{\beta}$= -0.067, p=0.027)  Non-significant association between life-course religious attendance and episodic memory ($\hat{\beta}$= -0.021, p=0.050)  Life-course religious attendance positively associated with self-rated memory ($\hat{\beta}$= 0.048, p=0.015)  Non-significant association between current religious attendance and working memory ($\hat{\beta}$= 0.057, p=0.056)  Non-significant association between current religious attendance and episodic memory ($\hat{\beta}$= -0.147, p=0.106)  Current religious attendance negatively associated with self-rated memory ($\hat{\beta}$= -0.061, p=0.034) |
| --- | --- | --- | --- | --- | --- | --- |

| Engelhardt et al., 2010 | Cross-sectional | n=22,949  52.49% women  Women: 63 years  Men: 62 years  Austria, Belgium, Denmark, France, Germany, Greece, Israel, Italy, Netherlands, Spain, Sweden, Switzerland  Community- dwelling  Exclusions: reported a stroke or cerebral vascular disease, Parkinson's disease or cancer, and Parkinson's disease took drugs for anxiety or depression or had been treated in a mental hospital or psychiatric ward | 1 score derived from participation in religious organization (yes vs. no) | Delayed recall to test recall  Higher score=better memory | Stochastic frontier model  Age, education, ppp-adjusted total gross income, chronic disease, social participation (e.g., volunteer work, sports participation), employment, physical activity, overweight/obese, smoking, drinking | Participation in religious organization associated with better recall ($\hat{\beta}$= -0.40, p<0.01)  In men, participation in religious organization associated with better recall ($\hat{\beta}$= -0.10, p<0.01)  In women, participation in religious organization associated with better recall ($\hat{\beta}$= -0.49, p<0.01) |
| --- | --- | --- | --- | --- | --- | --- |
| Hosseini et al., 2021 | Cross-sectional | n=24,669 for immediate recall test  n=24,439 for delayed recall test  50.9% female  63.0±10.2 years  Canada  Community- dwelling  Exclusions: resides in one of Canada’s three territories, or on federal First Nations reserves and other First Nations settlements in the provinces, were full-time members of the Canadian Armed Forces, were institutionalized individuals, cannot understand English or French, had signs of cognitive impairment at the recruitment interview | Single score derived from frequency of religious activity participation in last 12 months (daily to weekly, monthly to yearly, and no participation-reference) | 2 scores derived from:  Rey Auditory Verbal Learning Test (RAVLT) I to test immediate recall  RAVLT II to test delayed recall  Higher score=better memory | Multivariable linear regression  Sex, education, marital status, household income, province of residence, drinking, smoking, social participation (e.g., volunteering, recreation activity), social support, social; network, depression, chronic conditions, general health, Basic Activities of Daily Living and Instrumental Activities of Daily Living  Analyses dichotomized by age (<65 years and ≥65 years) | Non-significant association between daily-weekly religious activity participation (vs. No participation) and immediate recall in those <65 years ($\hat{\beta}$= 0.015; 95% CI= –0.057, 0.087)  Non-significant association between monthly-yearly religious activity participation (vs. No participation) and immediate recall in those <65 years ($\hat{\beta}$= 0.045; 95% CI= –0.002, 0.092)  Non-significant association between daily-weekly religious activity participation (vs. No participation) and immediate recall in those ≥65 years ($\hat{\beta}$= −0.037; 95% CI=–0.100, 0.026)  Non-significant association between monthly-yearly religious activity participation (vs. No participation) and immediate recall in those ≥65 years ($\hat{\beta}$= 0.018; 95% CI= –0.044, 0.080)  Non-significant association between daily-weekly religious activity participation (vs. No participation) and delayed recall in those <65 years ($\hat{\beta}$= 0.038; 95% CI= –0.034, 0.111)  Non-significant association between monthly-yearly religious activity participation (vs. No participation) and delayed recall in those <65 years ($\hat{\beta}$= 0.026; 95% CI= –0.023, 0.074)  Non-significant association between daily-weekly religious activity participation (vs. No participation) and delayed recall in those ≥65 years ($\hat{\beta}$= −0.048; 95% CI= –0.113, 0.018)  Non-significant association between monthly-yearly religious activity participation (vs. No participation) and delayed recall in those ≥65 years ($\hat{\beta}$= 0.021; 95% CI= –0.041, 0.083) |
| Lekhak et al., 2020 | Cross-sectional  Follow up time=12 years | n=1,135  57% women  67.5±10.1 years  USA  Community- dwelling | 1 private prayer score derived from praying privately in places other than church or synagogue (yes or no) | 2 episodic memory scores derived from:  Immediate recall test  Delayed recall test  Higher score=better memory | Generalized estimating equation Regression models  Age, meditation | Prayer positively associated with episodic memory ($\hat{\beta}$ =0.66, p<0.01) |
| Kraal et al., 2019 | Cross-sectional | n=16,089  59.20% women  68.44±10.15 years  USA  Community- dwelling | 3 scores derived from:  1) Frequency of religious attendance (0 (Not at all) to 4 (More than once a week))  2) Frequency of private prayer (0 (Never) to 7 (More than once a day))  3) Religious belief (higher values=stronger religious belief) | 2 scores derived from immediate and delayed recall tests to test:  1) Initial episodic memory performance  2) Rate of episodic memory change  3) Higher score=better memory | Single Structural Equation Model  Age, sex, education, wealth, chronic disease, depressive symptoms, social participation  Dichotomized by ethnicity (Non-Hispanic Black and Hispanic) | Private prayer positively associated with initial memory performance ($\hat{\beta}$= 0.014, p=0.007)  Religious attendance positively associated with initial memory performance ($\hat{\beta}$= 0.020, p<0.001)  Religious belief inversely associated with initial memory performance ($\hat{\beta}$= −0.023, p=0.004) |
| Kim et al., 2021 | Cross-sectional | n=164  75.6% female  78.21±5.3 years  South Korea    Community- dwelling  Exclusions: has schizophrenia, mental impairment, history of addiction such as alcohol or drug dependency, or other mental disorder, neurological diseases (e.g., stroke, brain tumor, Parkinson's disease, epilepsy, or severe head trauma) | 1 score derived from religion (yes or no) | 1 score derived from subjective memory function  Higher score=better memory | Stepwise multiple regression analysis  Age, education, sleep disorder, nutritional status, # of commodities, perceived health status, depression, self-esteem | Religion positively associated with subjective memory function ($\hat{\beta}$= 0.175, p=0.013) |
| Nelson et al., 2022 | Cross-sectional | n=2,716  57.2% female  50 years  USA  Community-dwelling | 1 score derived from frequency of religious service attendance (more than once a week, 2-4 times a month, once a month or less, or never (reference category)) | 1 score derived from RAVLT to test verbal memory | Multivariable linear regression  Age, sex, race, education, employment, marital status, body mass index, smoking status, drinking status, physical activity score, depressive symptoms, number of self-reported health conditions, study center, and social support at year 25, and religious services at year 2 | Religious attendance positively associated with verbal memory ($\hat{\beta}$= 0.17; 95% CI= 0.04, 0.30) |

CERAD=Consortium to Establish a Registry for Alzheimer’s Disease Assessment Packet; CPS=Cognitive Performance Scale; CHA=Community health assessment; WEL=Wellness assessment tool; FS=Functional supplement; RAVLT=Rey Auditory Verbal Learning Test
