# Supplementary material for: The association between religious participation and memory among middle-aged and older adults: A systematic review
Source: PLoS One. 2023 Aug 18;18(8):e0290279. doi: 10.1371/journal.pone.0290279 (PMC10437981; doi:10.1371/journal.pone.0290279)
Supplement: S7 Appendix — (DOCX) [file pone.0290279.s007.docx]

**S7 Appendix. Adapted Newcastle–Ottawa Scale for quality assessment of cross-sectional studies.**

| **Study** | **Selection** | | | | **Comparability** | **Outcome** | | **Total score** |
| --- | --- | --- | --- | --- | --- | --- | --- | --- |
|  | **Representativeness** **of the sample** | **Sample size** | **Ascertainment of exposure** | **Non-respondents** | **Comparability of different outcome groups** | **Assessment of outcome** | **Statistical test** |  |
| Engelhardt et al., 2010 | Non-random sampling, somewhat representative of average in target population  * | Not justified | Not validated instrument but described  * | No description of characteristics of responders and non-responders | Adjusted for age, education, ppp-adjusted total gross income, chronic disease, social participation (e.g., volunteer work, sports participation), employment, physical activity, overweight/obese, smoking, drinking  * * | Independent blind assessment  * | Statistical test is clearly described and appropriate, and measurement of the association is presented, including confidence intervals or p-values  * | 6 |
| Hill et al., 2020 | Random sampling, truly representative of average in target population  * | Not justified | Not validated instrument but described  * | No description of characteristics of responders and non-responders | Adjusted for  age, gender  education,  race/ethnicity, employment status,  wealth  early life relationship quality with mother,  marital status,  depressive symptoms,  self-rated health  * * | Independent blind assessment  * | Statistical test is clearly described and appropriate, and measurement of the association is presented, including confidence intervals or p-values  * | 6 |
| Hosseini et al., 2021 | Random sampling, truly representative of average in target population  * | Not justified | Not validated instrument but described  * | No description of characteristics of responders and non-responders | Adjusted for age, sex, education, marital status, household income, province of residence, drinking, smoking, social participation (e.g., volunteering, recreation activity), social support, social network, depression, chronic conditions, general health, Basic Activities of Daily Living and Instrumental Activities of Daily Living  * * | Independent blind assessment  * | Statistical test is clearly described and appropriate, and measurement of the association is presented, including confidence intervals or p-values  * | 6 |
| Jung et al., 2019 | Non-random sampling, somewhat representative of average in target population  * | Not justified | Validated instrument  * | No description of characteristics of responders and non-responders | Adjusted for age,  sex, years of education  * * | Independent blind assessment  * | Statistical test is clearly described and appropriate, and measurement of the association is presented, including confidence intervals or p-values  * | 6 |
| Kim et al., 2021 | Non-random sampling, somewhat representative of average in target population  * | Justified  * | Not validated instrument but described    * | No description of characteristics of responders and non-responders | Adjusted for age, education, sleep disorder, nutritional status, # of commodities, perceived health status, depression, self-esteem  * * | Independent blind assessment  * | Statistical test is clearly described and appropriate, and measurement of the association is presented, including confidence intervals or p-values  * | 7 |
| Kraal et al., 2019 | Non-random sampling, somewhat representative of average in target population  * | Not justified | Not validated instrument but described  * | No description of characteristics of responders and non-responders | Adjusted for age, sex, education, wealth, chronic disease, depressive symptoms, social participation  * * | Independent blind assessment  * | Statistical test is clearly described and appropriate, and measurement of the association is presented, including confidence intervals or p-values  * | 6 |
| Lekhak et al., 2020 | Random sampling, truly representative of average in target population  * | Not justified | Not validated instrument but described  * | No description of characteristics of responders and non-responders | Adjusted for age, gender, education, meditation, race, marital status, multimorbidity, age*prayer  * * | Independent blind assessment  * | Statistical test is clearly described and appropriate, and measurement of the association is presented, including confidence intervals or p-values  * | 6 |
| Strout et al., 2015 | Non-random sampling, somewhat representative of average in target population  * | Not justified | Validated instrument  * | No description of characteristics of responders and non-responders | Adjusted for age,  Education,  Social wellness,  intellectual wellness, physical wellness, emotional wellness  * * | Independent blind assessment  * | Statistical test is clearly described and appropriate, and measurement of the association is presented, including confidence intervals or p-values  * | 6 |
| Nelson et al., 2022 | Random sampling, truly representative of average in target population  * | Not justified | Not validated instrument but described    * | No description of characteristics of responders and non-responders | Adjusted for age, sex, race, education, employment, marital status, body mass index, smoking status, drinking status, physical activity score, depressive symptoms, number of self-reported health conditions, study center, social support, religious services  * * | Independent blind assessment  * | Statistical test is clearly described and appropriate, and measurement of the association is presented, including confidence intervals or p-values  * | 6 |

Each study can earn a maximum of 8 stars (*selection*: 4 stars, *comparability*: 2 stars, *outcome*: 2 stars). In the *comparability* section, a study is awarded 1 star if it controls for age, the most important factor, or an additional factor (sex, gender, education, or marital status) and 2 stars for both [26].
