# Supplementary material for: The association between religious participation and memory among middle-aged and older adults: A systematic review
Source: PLoS One. 2023 Aug 18;18(8):e0290279. doi: 10.1371/journal.pone.0290279 (PMC10437981; doi:10.1371/journal.pone.0290279)
Supplement: Attachment — Submitted filename: Response_to_Reviewers.docx [file pone.0290279.s009.docx]

**Reviewer 1**

| **Comment** | **Response** |
| --- | --- |
| Although the decision not to conduct a meta-analysis due to the heterogeneity of results in the included articles is understandable, a main figure that effectively conveys the characteristics of the reviewed studies, such as consistency in positive association, small effect size, and wide confidence interval, is necessary. I suggest that the authors consider presenting an adequate figure, such as a comparison of effect size and sample size, to effectively convey the characteristics of the reviewed studies. | We revised the forest plot in Appendix S6; the dots representing the point estimates of the regression coefficients were resized to be proportionate to the sample sizes of the included articles. This plot was also named Figure S6.1 because we added a second plot – Figure S6.2 – to Appendix S6. This second plot is a scatterplot showing the point estimates of the regression coefficients on the x-axis and the sample sizes on the y-axis. Please see our response to the reviewer’s second comment below for an explanation of how we worked these two plots into the text. |
| As the authors noted, the confidence intervals for four point estimates were wide, making it difficult to determine the magnitude of the effect. As a systematic review, the source of such large variability should be noted. One potential source is the sample size, as most studies did not perform sample size calculations. The authors could compare the sample sizes of the studies in the main text, although the exact numbers are specified in the supplementary materials. This would provide valuable information and help readers better understand the results and limitations of the reviewed studies. | We followed the reviewer’s suggestion and compared the sample sizes. The comparisons were made specifically to the regression coefficients and widths of the confidence intervals. Drawing upon Figures S6.1 and S6.2 as visual aids, we added the following text to the results section:  “Two of the three articles with the largest sample sizes [15,32] had the smallest $\hat{\beta}$s and among the narrowest confidence intervals, although the article with the largest sample size [30] had the second largest $\hat{\beta}$ and the third widest confidence interval (Figure S6.1). Three of the four articles with the smallest sample sizes had the widest confidence intervals [28,31,33]. Three of the five articles with $\hat{\beta}s$between 0.0 and 0.1 [15,32,36] had sample sizes above 5000, while three of the four articles with the largest $\hat{\beta}s$ (0.38 to 0.50) had sample sizes of 1,135 or less [28,31,33] (S6 Appendix; Figure S6.2).” |

**Reviewer 2**

| **Comment** | **Response** |
| --- | --- |
| The hypothesis tested in this manuscript consists of heterogeneous and confounded exposures and outcomes. Religious practice/ spirituality includes different religions/ and practices which made the exposure studied heterogeneous. Also, the outcome (different types of memory measures) is also heterogeneous and can be confounded by normal aging, neurodegeneration, sleep disorders, psychiatric disorders, neurologic disorders etc. It is not clear if the included studies accounted for these clinical diagnoses in their study design and thus the conclusion from the systematic review may not be accurate. | We thank the reviewer for this comment. The authors of the included articles accounted for these items by controlling for them as covariates or excluding persons with these conditions from the studies. For example, Engelhart et al. excluded those who reported a stroke, cerebral vascular disease, Parkinson's disease, or those who were taking medications for anxiety or depression, or persons who had received treatment in a psychiatric unit; Hill et al. controlled for depressive symptoms; Hosseini et al. excluded individuals with cognitive impairment and controlled for depressive symptoms. Given that we only acknowledged when authors accounted for these items as covariates, we have added information on exclusion criteria to the S5 Appendix.  We acknowledged the heterogeneity introduced by different eligibility criteria by modifying the second sentence in the section called ‘Sources of Heterogeneity’: “Heterogeneity manifested itself in the statistical reporting of results, assessments of exposure, mixes of covariates in regression models, and differences in the eligibility criteria for inclusion of participants.” |
| One main confounder is loneliness. Loneliness has been strongly associated with increased risk for dementia (which affects memory typically). Religious practices usually increase sense of community and decrease loneliness, and this could be what drives the association in positive studies. Please review: https://n.neurology.org/content/98/13/e1337 | We expanded the second-to-last paragraph of the discussion to incorporate this point and we cited the publication mentioned by the reviewer (our reference # 49). The expanded paragraph, with our changes or additions underlined, is as follows:  “Other items to explore would be the potential mediating or moderating roles of factors such as depression, social support, or loneliness in the relation between religion and memory. Depression has strong associations with both religion and memory, particularly among older adults, though its moderating or mediating role has not been thoroughly explored in this area. Religious involvement has been linked to hope, resiliency, and the ability to cope with adversities among depressed persons [45], and depression has been found to increase the risk for impaired memory [46]. Functional social support has been shown to be positively associated with memory [47], and social isolation/loneliness have been shown to be inversely associated with memory [48]. Given the links between memory function and major neurocognitive disorder, these forms of social connectedness may also be associated with dementia [47,49]. Religious involvement may promote functional social support and decrease loneliness/social isolation [50,51].” |
| My issue is not with the methods/ results, my issue is with the hypothesis itself. A very heterogeneous “exposure” is been studied for its association with a very heterogeneous outcome “different memory measures” in participants with unknown clinical data and in cross sectional studies that are biased and weak. Again, loneliness could be the main mediating factor here (if the association stands). Thus, the results and conclusion were predictably non-decisive and just showed the need for more and better research. Thus, I am not sure how much this manuscript adds to the literature, except that it provides an updated summary of weak studies. | We agree with the reviewer that loneliness could be an important mediator of associations between religion and memory, although none of the included articles examined mediation by loneliness, nor did they consider loneliness in any other capacity. We added the following text to the “Summary of Evidence” in the Discussion to address this limitation of the literature: “Furthermore, none of the studies assessed mediating factors such as loneliness and, therefore, provided limited information on the underlying components of the mechanism of association between religion and memory.”  We also added the following text to “Future Recommendations and Conclusion” in the Discussion: “Other items to explore would be the potential mediating or moderating roles of factors such as depression, social support, or loneliness in the relation between religion and memory. Depression has strong associations with both religion and memory, particularly among older adults, though its moderating or mediating role has not been thoroughly explored in this area. Religious involvement has been linked to hope, resiliency, and the ability to cope with adversities among depressed persons [45], and depression has been found to increase the risk for impaired memory [46]. Functional social support has been shown to be positively associated with memory [47], and social isolation/loneliness have been shown to be inversely associated with memory [48]. Given the links between memory function and major neurocognitive disorder, these forms of social connectedness may also be associated with dementia [47,49]. Religious involvement may promote functional social support and decrease loneliness/social isolation [50,51].” |

**Reviewer 3**

| **Comment** | **Response** |
| --- | --- |
| 1 Selection Bias: The authors note a lack of comparison between respondents and non-respondents in the included studies, which prevents a full understanding of selection bias. This lack of comparison could skew the conclusions of the review, as it could fail to account for any systematic differences between these two groups.  2 Lack of Longitudinal Studies: All studies reviewed were cross-sectional, which means the researchers could not determine cause-and-effect relationships, only correlations. Longitudinal studies would have allowed for a more nuanced understanding of how religious involvement affects memory over time.  3 Lack of Diversity in Faiths: The sample of reviewed studies was predominantly set in Euro-American contexts, likely skewing towards Christian populations. This limits the generalizability of the findings to other religious groups.  4 Heterogeneity of Exposure and Outcome Measures: The various ways of measuring exposures (religion) and outcomes (memory) in the included studies made it difficult to ascertain specific correlations. This high level of heterogeneity also prevented the authors from conducting a meta-analysis. | These are all valid points that we addressed in the discussion. |
| Expanded Inclusion Criteria: The authors could consider including non-English language studies to diversify the cultural and religious contexts of their data. This could potentially yield more nuanced and globally applicable insights. | Much of the research on religion and memory is not focused specifically on one religion versus another. We simply speculated that the Euro-American settings of most included studies likely yielded predominantly Christian samples. As a result, we advised readers to exercise caution when applying the results to non-Christian settings. Given the dearth of English-language research on the topic, we are unlikely to find a substantive body of literature in other languages to enable meaningful comparisons across different cultural and religious contexts. |
| Conduct a Meta-Analysis: If possible, it would be beneficial to standardize or categorize the different exposure and outcome measures to allow for a meta-analysis. This could provide more quantitative insights into the magnitude and direction of the effect of religion on memory. | Ideally, we would have liked to conduct a meta-analysis, but substantive differences across the included articles prevented us from doing so. The exposure and outcome measures were simply too disparate to permit any sort of combination. |
| Collaboration for New Research: Given the identified gaps in longitudinal data and diversity of faiths, the authors could collaborate with other researchers or institutions to conduct new, well-designed longitudinal studies. These should aim to fill the highlighted gaps, including various religious denominations and more diverse geographical and cultural contexts. | Thank you for this suggestion. |
| Consideration of Other Variables: The authors might consider the potential impact of other variables such as age, socioeconomic status, and education. Accounting for these variables could help to explain some of the observed associations between religion and memory. | We created a subheading: “Consideration of the impact of other variables on the association” in the Results section after the Risk of bias paragraph, and added the following paragraph:  “We also assessed whether any other variables such as age, gender and socioeconomic status had an impact on the association between religious participation and memory in our review. Out of all the included studies, only Lekhak et al. [31] addressed the impact of other variables, namely age and gender, on this association. In the study, it was found that after adjusting for gender, more women reported using prayer and had a higher episodic memory score compared to men (β = 1.11, p < .001) [31]. The authors also noted a slightly higher episodic memory score among those who pray as they become older (β = 0.04, p < .05) [31].” |
| In-depth Analysis of Non-significant Results: It could be helpful to discuss the non-significant results in more depth. This could potentially offer important insights into the circumstances or factors that might mitigate the association between religion and memory. | Under the subheading “Verbal episodic memory & short-term/immediate and delayed recall memory” in the Results section, we added the following text to the fifth paragraph about Hill et al.’s study: “The non-significant associations in the study may be attributed to the small sample of 516 participants.”  However, we could not find any patterns that explained why some articles may have reported non-significant findings, while other articles reported significant findings. For example, see our response to the second comment from reviewer 1. |
| In order to create a meaningful forest plot, the authors have combined various exposure and outcome measures to generate single effect estimates. For instance, they used the 'aggregate' function in R’s metafor package to combine the regression coefficients for three types of religion into one effect estimate in the study by Kraal et al. This approach is valid but, as the authors have noted, it introduces an element of assumption, specifically the assumption of a within-study correlation across regression coefficients of 0.5. In the absence of actual data, this assumption might be reasonable, but it also introduces a degree of uncertainty into their results.  Additionally, the authors have made some assumptions to deal with missing data, which is another common problem in systematic reviews. Specifically, they've estimated standard errors for studies that only reported p-values but not standard errors or confidence intervals. They've done this by conservatively assuming the actual p-value of the findings was 0.001 and using the ‘se.from.p’ function in R’s dmetar package. This approach seems prudent, but it does rely on an assumption that may or may not be accurate.  Lastly, the authors standardized the direction of effect in the forest plot by taking the absolute values of negative regression coefficients when higher scores on memory measurement instruments indicated poorer outcomes. This ensures that a positive regression coefficient consistently indicates a positive association between religion and memory in the plot. It's a reasonable approach to standardize the interpretation of the results, but it's worth noting that it involves manipulating the original data, which could potentially obscure some of the nuances of the original findings.  In summary, while the authors' approach to dealing with the heterogeneity of the studies and missing data is innovative and careful, it does introduce a degree of uncertainty into their results. The use of assumptions in their analysis means that the true effect sizes might be somewhat different from what they've reported. Future research in this area would benefit from more consistent reporting of statistical results and measures in primary studies, which would enable a more precise and confident synthesis of the evidence. | We agree that the accuracy of the forest plot depends on the validity of the assumptions and we added the following sentences to the section on ‘strengths and limitations of the review’: “To assist in presenting a comprehensive snapshot of the results, we developed a forest plot that relied on certain assumptions about correlations across regression coefficients, p-values, and directions of effect (described under ‘summary forest plot’ above). The accuracy of the plot depends on the validity of these assumptions.” |
