# Supplementary material for: The association between religious participation and memory among middle-aged and older adults: A systematic review
Source: PLoS One. 2023 Aug 18;18(8):e0290279. doi: 10.1371/journal.pone.0290279 (PMC10437981; doi:10.1371/journal.pone.0290279)
Supplement: Attachment — Submitted filename: Response_to_Reviewers.docx [file pone.0290279.s010.docx]

**Reviewer 1**

| **Comment** | **Response** |
| --- | --- |
| One major concern I raised in my initial review was that the “take-home message throughout the main text and especially for figures is hard to grasp”. Unfortunately, I did not find any specific response addressing this issue in the "response to reviewers." I kindly request the authors to reconsider the clarity of the take-home message and ensure that it is effectively conveyed in the main figures. While the authors have revised the supplementary figure, I still strongly recommend incorporating the relevant contents of Fig. S.6 into the main figure to better present their findings. One possible approach could be to include Fig. S.6 into the Figure 3. | We do not have a ‘Figure 3’ in the manuscript. Presumably, the reviewer was referring to Figures S6.1 and S6.2. After some testing of combined figures, we found we could not develop a single figure that amalgamated and clearly depicted all elements of both figures. Therefore, we chose the keep the figures separate. However, to enhance interpretation of the figures, we added a dashed regression line to Figure S6.2, which showed an inverse relation between effect size and sample size. We added the following sentence to the text on p. 11 after inserting the regression line into the figure: “This suggests an inverse relation between effect size and sample size, as shown by the downward-sloping and dashed line in Figure S6.2.”  We added the following ‘take-home’ message to the final paragraph of the conclusion (pp. 18-19) and integrated commentary about the figures into this message: “Our systematic review provided a comprehensive overview of research on the roles of religion and memory in later life. Most included studies reported positive associations between religion and memory [14, 30, 28, 33, 31, 36], as shown by the summary effects in Figure S6.1. However, we could not draw definitive conclusions about the true direction or magnitude of effect. This was because of wide confidence intervals in some instances, moderate levels of risk of bias in eight of nine studies, and cross-sectional designs. Further, the included studies contained some important research gaps by not addressing whether different faiths, sex/gender and depression affected the association of interest. Additionally, many studies did not adequately handle prognostic factors, as only four studies excluded persons with neurological conditions [15, 28, 30, 33] and five studies adjusted for depressive symptoms in regression analysis [14, 15, 32, 33, 34] (S5 Appendix). While stronger evidence from further studies is needed to fill current research gaps, the review highlighted the state of the published literature on this topic.” |
| I appreciate the authors' efforts in responding to the “specific comments”, especially in terms of clarifying the description of confidence intervals, the scale of β estimates, and the sample size. These revisions indeed support the conclusion “The summary effects in the forest plot showed a positive association between religion and memory overall, though small effect sizes in some instances and wide confidence intervals in other instances limited our ability to draw conclusions about the magnitude of the true effect.”. | Thank you. |
| In Figure S6.2, I noticed that the x-axis label indicates "(95% confidence interval)”, despite there is no confidence interval displayed. | We corrected the x-axis label and removed “(95% confidence interval)”. |

**Reviewer 2**

| **Comment** | **Response** |
| --- | --- |
| The authors in their reply mentioned:  "Engelhart et al. excluded those who reported a stroke, cerebral vascular disease, Parkinson's disease, or those who were taking medications for anxiety or depression, or persons who had received treatment in a psychiatric unit; Hill et al. controlled for depressive symptoms; Hosseini et al. excluded individuals with cognitive impairment and controlled for depressive symptoms. Given that we only acknowledged when authors accounted for these items as covariates, we have added information on exclusion criteria to the S5 Appendix."  I think this should be clearly mentioned in the discussion section, and they should add that most studies did not account for neurocognitive disorders, dementias or neurodegenerative disorders that clearly affect memory (which include Alzheimer, hippocampal sclerosis, vascular related cognitive decline, FTD, LBD, PD etc). This missing information makes the results of any of the included studies uninterpretable. The authors should clearly say that and clearly say that the conclusions of the meta-analysis is very weak and cannot be accurately trusted knowing that the included studies all have very serious flaws. | We revised the final paragraph of the discussion section (pp. 18-19) to include a ‘take-home’ message, as requested by Reviewer # 1. In this message, we added the following text: “Additionally, many studies did not adequately handle prognostic factors, as only four studies excluded persons with neurological conditions [15, 28, 30, 33] and five studies adjusted for depressive symptoms in regression analysis [14, 15, 32, 33, 34] (S5 Appendix)”. We were also clear to point out that readers could not take the results portrayed in Figure S6.1 at face value (p. 18): “Most included studies reported positive associations between religion and memory [14, 30, 28, 33, 31, 36], as shown by the summary effects in Figure S6.1. However, we could not draw definitive conclusions about the true direction or magnitude of effect.” After these two sentences, we elaborated on our conclusion and included the aforementioned text about the prognostic factors. We also wrote (p. 19): “While stronger evidence from further studies is needed to fill current research gaps, the review highlighted the state of the published literature on this topic.” |
